# Supplementary material for: Groundwater Chemistry and Blood Pressure: A Cross-Sectional Study in Bangladesh
Source: Int J Environ Res Public Health. 2019 Jun 28;16(13):2289. doi: 10.3390/ijerph16132289 (PMC6651438; doi:10.3390/ijerph16132289)
Supplement: Supplementary file 1 [file ijerph-16-02289-s001.pdf]

Groundwater chemistry and blood pressure: a cross-sectional study in Bangladesh

Table S1: Bangladesh Demographic and Health Survey 2011

Survey Name	Bangladesh Demographic and Health Survey
Survey year	2011
Sampling method	Nationally representative covering the entire population residing in non-institutional dwelling units.
Total clusters	600
Total households	Average 30 households per cluster.
Report	https://dhsprogram.com/pubs/pdf/FR265/FR265.pdf
Implementing organization	Conducted under the authority of the National Institute of Population Research and Training (NIPORT) of the Ministry of Health and Family Welfare and implemented by Mitra and Associates of Dhaka.
Funding source	ICF International provided financial and technical assistance for the survey through USAID/Bangladesh
Data source/website	https://dhsprogram.com/Data/ [permission required to access data]

Groundwater chemistry and blood pressure: a cross-sectional study in Bangladesh

Table S2: British Geological Survey and Department of Public Health Engineering well survey in Bangladesh

Survey name	British Geological Survey and Department of Public Health Engineering Survey
Survey year	2000
Sampling method	Across all districts and sub-districts of Bangladesh except for three districts of Chittagong.
Total wells	3,534
Chemicals tested	Arsenic, sodium, potassium, calcium, magnesium, boron, barium, cobalt, chromium, copper, iron, lithium, manganese, phosphorus, silicon, sulfate, zinc, strontium, vanadium
Method for analysis	Arsenic by hydride generation-atomic fluorescence spectrometry (HG-AFS) in the UK but some of the early samples collected in the Phase I survey were analysed by hydride generation-ICP-AES. Additional elements in the survey samples were measured by ICP-AES .
Laboratory used for analysis	British Geological Survey (BGS) laboratories
Report	http://www.bgs.ac.uk/research/groundwater/health/arsenic/Bangladesh/reports.html
Implementing organization	The Department of Public Health Engineering (DPHE) and BGS
Data source/website	http://www.bgs.ac.uk/research/groundwater/health/arsenic/Bangladesh/data.html

Groundwater chemistry and blood pressure: a cross-sectional study in Bangladesh

Table S3: Blood pressure (mmHg) among different categories of participants

Characteristics	Systolic blood pressure mean (95% CI) mm Hg	Diastolic blood pressure mean (95% CI) mm Hg
Arithmetic mean	118.7 (118.0, 119.4)	77.9 (77.5, 78.5)
Geometric mean	116.9 (116.4, 117.4)	79.8 (79.2, 80.3)
Age categories		
≥35 — 50 years	114.1 (113.5, 114.8)	77.7 (77.2, 78.2)
≥50 — 65 years	120.2 (119.1, 121.3)	78.2 (77.6, 78.8)
≥ 65 years	129.7 (127.9, 131.5)	78.1 (77.4, 78.9)
Sex		
Male	116.0 (115.2, 116.8)	76.3 (75.8, 76.8)
Female	121.4 (120.5, 122.3)	79.5 (79.0, 80.0)
BMI categories		
Underweight (<18.5 kg/m^2)	115.1 (113.8, 116.3)	74.2 (73.6, 74.8)
Normal weight (≥18.5 to <25 kg/m^2)	118.5 (117.8, 119.3)	78.3 (77.9, 78.9)
Overweight (≥25 to <30 kg/m^2)	123.8 (122.2, 125.3)	83.3 (82.4, 84.2)
Obese (≥ 30 kg/m^2)	127.4 (123.7, 131.1)	85.7 (83.8, 87.6)
Education categories		
No institutional education	119.9 (118.9, 120.8)	77.4 (76.9, 78.0)
Primary level	116.7 (115.7, 117.7)	77.5 (76.8, 78.1)
Secondary level	117.5 (116.4, 118.7)	78.9 (78.2, 79.6)
College level or higher	121.4 (119.7, 123.0)	81.0 (80.1, 81.9)

Groundwater chemistry and blood pressure: a cross-sectional study in Bangladesh

Table S4: Association of groundwater chemicals with blood pressure when wells within 2.3 kilometers of BDHS clusters were considered.

Groundwater chemicals	Models	Systolic blood pressure			Diastolic blood pressure		
		GMR	Lower 95% CI	Upper 95% CI	GMR	Lower 95% CI	Upper 95% CI
Na	Model 1	0.995	0.989	1.000	0.991	0.984	0.999
	Model 2	0.999	0.994	1.003	0.993	0.987	0.999
	Model 3	0.995	0.987	1.003	0.987	0.979	0.994
Ca	Model 1	1.000	0.991	1.009	0.999	0.990	1.007
	Model 2	0.992	0.981	1.003	0.994	0.984	1.005
	Model 3	0.993	0.972	1.015	0.996	0.977	1.016
Mg	Model 1	0.991	0.983	0.998	0.989	0.982	0.997
	Model 2	0.993	0.986	1.001	0.993	0.985	1.000
	Model 3	0.985	0.968	1.003	0.988	0.973	1.004
K	Model 1	0.997	0.989	1.004	0.995	0.987	1.002
	Model 2	1.000	0.994	1.006	0.998	0.992	1.004
	Model 3	1.004	0.996	1.013	1.001	0.993	1.008
Fe	Model 1	0.997	0.989	1.005	0.998	0.990	1.006
	Model 2	0.996	0.988	1.005	0.998	0.990	1.006
	Model 3	0.998	0.988	1.007	0.999	0.990	1.008
Si	Model 1	0.993	0.985	1.001	0.997	0.990	1.004
	Model 2	0.995	0.988	1.003	1.001	0.994	1.007
	Model 3	0.993	0.985	1.002	0.997	0.990	1.004
SO4	Model 1	1.000	0.989	1.011	0.998	0.986	1.010
	Model 2	0.999	0.992	1.007	1.001	0.993	1.009
	Model 3	0.991	0.966	1.016	1.007	0.984	1.031
As	Model 1	0.993	0.987	1.001	0.993	0.986	0.999
	Model 2	0.998	0.991	1.005	0.997	0.990	1.004
	Model 3	0.999	0.991	1.007	0.999	0.992	1.006
Ba	Model 1	1.004	0.991	1.017	1.004	0.992	1.017
	Model 2	0.996	0.982	1.009	0.995	0.982	1.008
	Model 3	1.010	0.991	1.030	1.007	0.990	1.024
Zn	Model 1	1.008	0.979	1.038	1.000	0.976	1.023
	Model 2	1.003	0.971	1.035	0.995	0.968	1.023
	Model 3	0.996	0.968	1.024	0.985	0.961	1.009
Mn	Model 1	1.006	1.001	1.010	1.001	0.996	1.006
	Model 2	1.006	0.999	1.011	1.004	0.999	1.010
	Model 3	1.008	1.003	1.013	1.006	1.001	1.011

Groundwater chemistry and blood pressure: a cross-sectional study in Bangladesh

Table S5: Pooled estimates from the meta-analysis of stratified analysis based on geological unit

Blood pressure	Model 1<br>GMR (95% CI)	Model 2<br>GMR (95% CI)	Model 3<br>GMR (95% CI)
**Systolic BP**	0.995 (0.989, 1.001)	0.995 (0.989, 1.000)	0.977 (0.957, 0.997)
**Diastolic BP**	0.994 (0.988, 1.000)	0.995 (0.989, 1.000)	0.964 (0.944, 0.983)
